# Laser-Based Synthesis of Au Nanoparticles for Optical Sensing of Glyphosate: A Preliminary Study

**DOI:** 10.3390/mi11110989

**Published:** 2020-10-31

**Authors:** Antonella Laura Sortino, Maria Censabella, Gabriella Munzi, Simona Boninelli, Vittorio Privitera, Francesco Ruffino

**Affiliations:** 1 CNR-IMM (Consiglio Nazionale delle Ricerche-Istituto per la Microelettronica e i Microsistemi) via S. Sofia 64, 95123 Catania, Italy; anto87.111@gmail.com (A.L.S.); simona.boninelli@ct.infn.it (S.B.); vittorio.privitera@imm.cnr.it (V.P.); 2 Dipartimento di Fisica e Astronomia “Ettore Majorana”, Università di Catania, via S. Sofia 64, 95123 Catania, Italy; maria.censabella@ct.infn.it; 3 Dipartimento di Scienze Chimiche, Università di Catania, Viale Andrea Doria 6, 95125 Catania, Italy; gabryamunzi@tiscali.it

**Keywords:** glyphosate, pulsed laser ablation, gold nanoparticles, colorimetry, optical sensing

## Abstract

Nowadays, Au nanoparticles (AuNPs) capture great interest due to their chemical stability, optical properties, and biocompatibility. The success of technologies based on the use of AuNPs implies the development of simple synthesis methods allowing, also, the fine control over their properties (shape, sizes, structure). Here, we present the AuNPs fabrication by nanosecond pulsed laser ablation in citrate-solution, that has the advantage of being a simple, economic and eco-sustainable method to fabricate colloidal solutions of NPs. We characterized the stability and the absorbance of the solutions by Ultraviolet-Visible (UV-Vis) spectroscopy and the morphology of the AuNPs by Transmission Electron Microscopy. In addition, we used the AuNPs solutions as colorimetric sensor to detect the amount of glyphosate in liquid. Indeed, glyphosate is one of the most widely used herbicides which intensive use represents a risk to human health. The glyphosate presence in the colloidal AuNPs solutions determines the aggregation of the AuNPs causing the change in the color of the solution. The variation of the optical properties of the colloidal solutions versus the concentration of glyphosate is studied.

## 1. Introduction

In recent years, the advent of nanotechnology has allowed a widespread use of nanostructures in the industrial, biological, biomedical, fuel cells, and optical fields, this thank their unique chemical and physical properties related to large surface/volume ratio respect to bulk materials [1,2,3,4]. Particular interest is directed towards metallic nanoparticles and especially noble metal nanoparticles (Au, Pt, Pd, Ag, Rh, Ru, Ir, and Os) that show unique properties, such as resistance to corrosion and oxidation, high melting point, non-reactiveness, and high ionization energy [5,6,7,8,9,10,11,12,13]. Compared to non-noble metals, they show antibacterial action and, in the field of catalysis, they increase the yield of redox reactions [14,15,16,17,18,19]. In addition, under excitation by electromagnetic radiation, Au and Ag present localized surface plasmon resonance (LSPR) [2,11,20,21,22], i.e., collective oscillations of free-electrons. Consequently, a strong absorption band appears in some region of the electromagnetic spectrum, which lead to unique properties, such as strong resonant absorption/scattering, intense field enhancement, ultrasensitive biosensing, which makes them highly interesting for multiple purposes [23,24].

Nevertheless, the success of such technologies implies knowledge and control over the nanostructures’ properties (shape, sizes, structure and crystallinity) and therefore the development of simple, versatile, low cost techniques for their production allowing such a fine control.

Au nanoparticles (AuNPs) can be synthetized by chemical or physical methods. Chemical approaches as, for example, those based on the reduction of a precursor salt with a reducing agent (i.e., Turkevich method [25]) are powerful methods to produce AuNPs in solution being cheap, easy, eco-friendly, and high-throughput, even if these methods often lead to NPs surface contamination. In fact, some residual anions and the reducing agents could remain on the surface of the synthesized nanoparticles and, depending on the application, they should be removed by post-preparation process, which can impact on cost time and money [26]. From another hand, the nanotechnology revolution needs the continue development of alternative nanofabrication approaches matching the low-cost, simplicity, eco-friendly, high-throughput requirements. Hence, in addition to chemical-based methods for the production of AuNPs in solution, also physical-based fabrication methods are continuously designed and developed so to enhance the versatility strength of the nanofabrication approaches. As a consequence, depending on the final NPs applications, requiring specific NPs characteristics, the most suitable fabrication approach can be chosen. In this sense, a physical-based technique for the direct production of NPs in solution, often alternative to chemical-based methods is the Pulsed Laser Ablation in Liquid environment (PLAL). Rather than alternative to chemical based methods, PLAL can be recognized as a physical-based approach for the production of NPs in solution completing the techniques based on the reduction of a precursor salt with a reducing agent so enhancing the possibility to produce designed NPs for specific applications. PLAL is, nowadays, widely recognized as a competitive, versatile, low cost, and green technique that allows to fabricate bare or capped surface nanoparticles with desired physicochemical and structural properties [27]. A big advantage of laser-generated NPs is the exclusion of toxic substances or by-products, since chemical precursors are not needed [28]. In addition, the nanoparticles formation by PLAL is influenced by different laser parameters (such as wavelength, pulse duration, repetition rate and fluence) and allows the production of NPs from any base material (metal, alloy, semiconductor, oxides, nitrides, or carbides). Also, the liquid environments play an important role: Indeed, changing the solvent is possible to produce NPs with different surface chemistry, surface charge or sizes [28,29]. So, playing on all these parameters of PLAL, nanoparticles with the desired shape, size, and compositions can be obtained. In particular, compared to chemical synthesis methods of AuNPs based on the reduction of a precursor salt with a reducing agent, the PLAL technique is, surely, cost-competitive [28]: The most expensive part of this setup is the laser system; all the other costs are almost negligibly small, but also depend on the target you use. Regarding laser ablation of Au (purity of 99.99%) in water the Au target mostly influences the price. However, for laser ablation not the whole target is, generally, transformed to NPs, but the remains can be recycled by molding a new target. By laser ablation of a target, the value of the material can be multiplied up to three orders of magnitude producing several liters of the NPs colloidal solution. On the other hand, wet chemically AuNPs synthesis requires expensive Au salt that is mixed with a reducing agent and the excessive amount of organic reactants and by-products forming ligands on the NPs’ surface, depending on the application, should be removed costing time, energy, and money. In this regard, purity and simplicity of the PLAL techniques have multiple values.

During PLAL, a beam laser is focused by an optical system on a solid target in liquid environment, then the radiation absorbed by the target leads to the formation of an expanding plasma plume, which contains the ablated material. When the plasma cools down, heat is released environment and transformed into hot vapor. So, an oscillating cavitation bubble is formed, that contains both the ablated matter and the liquid vapor. Later, the cavitation bubble collapses and the particles are released into the solvent, forming a nanoparticles colloidal solution [28,30,31]. The produced NPs have highly active surface allows a better biocompatibility for in vivo applications or their functionalization with the desired ligands for their use as nanostructured sensors [26,32,33,34,35].

Recently, various nanostructured systems have been explored as sensing elements for the detection of glyphosate content in various systems [36,37,38,39,40,41,42] and metallic nanoparticles are widely used in this regards in particular to fabricate colorimetric sensors [43,44,45,46,47]. Glyphosate (N-(phosphonomethyl)glycine) is an organophosphorous pesticides (OPPs) which is used worldwide for weed and vegetation control in cultures and gardens. The intensive use of glyphosate represents a risk to human health: Indeed, recent studies suggest that it affects cell cycle regulation, determines a loss of fertility in men, and increases tumor incidence. In addition, the OPPs inhibit the activity of acetylcholinesterase, the enzyme required to breakdown the neuro-transmitter acetylcholine at cholinergic synapses, affecting the human nervous system [48]. Residuals of glyphosate were found in food (cereals) and in natural waters [49,50,51,52]. For these reasons, the request to design fast and cost-effective methods for the detection of glyphosate content in water samples is continuously increasing.

The use of colloidal solutions of Au nanoparticles as a colorimetric sensor meets this request, since it is a simple, low cost and quickly detectable method, even with the naked eye. Indeed, in this technique, by coherently customizing the functional groups of the nanoparticles, the target analyte binds to the particles creating a controlled aggregation; this effect changes the characteristic absorption spectrum of the solution and therefore its c, letting analyte detection [45,49].

In recent years, for example, R. E. De Góes et al. [46,47] used citrate-stabilized laser ablated silver nanoparticles for glyphosate detection. Starting from these results and taking into account the simplicity of the laser ablation nanoparticles production technique and the easy and speed method for glyphosate sensing, in this work, we aim to extend the investigation to laser ablation-fabricated AuNPs reporting some additional insights. In particular, we report on the fabrication of AuNPs by PLAL in citrate solution. Sodium citrate was used as capping agent to prevent AuNPs aggregation. We characterized the stability and the absorbance of the solutions by UV-Vis (UltraViolet-Visible) measurements and the morphology of the NPs by Transmission Electron Microscopy (TEM). Moreover, as a preliminary study towards in-depth future investigations, we report on the use of the citrate-capped AuNPs in water solution to detect the amount of glyphosate in water exploiting the color change of the solutions and their absorbance spectrum due to glyphosate’s presence. The limit of detection (LOD) and sensitivity value for glyphosate detection in water are quantified.

## 2. Materials and Methods

### 2.1. Synthesis of Colloidal Nanoparticles

**Figure 1:**
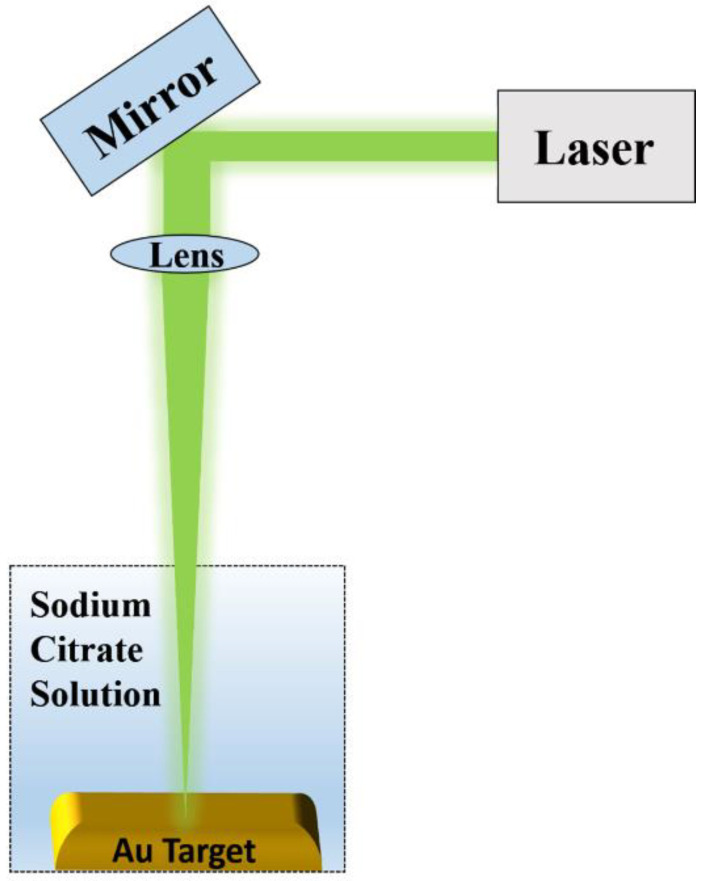
Schematic of the pulsed laser ablation process used for the synthesis of colloidal nanoparticles (NPs).

Colloidal solutions of gold nanoparticles have been synthetized by pulsed laser ablation from a metal target made by Au (thickness of 1.0 mm, purity of 99.999%). The scheme of the pulsed laser ablation process is shown in Figure 1. In particular, a beam of Nd: Yttrium Aluminum Garnet YAG Laser (Quanta-ray PRO-Series pulsed Nd: YAG laser (Spectra Physics, Santa Clara, CA, USA), 10 ns pulse, λ = 1064 nm, fluence 5 J/cm^2^, frequency of 10 Hz, laser spot 2 mm) was focalized by means of a 20 cm focal length lens on the target at the bottom of a teflon vessel, filled with 8 mL of citrate solution 0.1 mM. A stock citrate solution was prepared dissolving 1.47 g of sodium citrate (CAS number 6132043, www.sigmaaldrich.com) in 50 mL of deionized Milli-Q water (resistivity 18 MΩ cm) and stored at standard temperature (25 °C). After the stock solution was diluted down to 0.1 mM by adding deionized Milli-Q water. Three samples were prepared by changing the ablation times to 5, 8, and 12 min.

During the irradiation of the target, the solution’s color becomes red wine, going from light red (5 min of ablation time) to dark red (12 min). The color depends on the scattering and absorption contributions. Indeed, the solution of gold nanoparticles strongly absorbs light at 520 nm (green light) and 450 nm (blue light), while red light (~700 nm) is reflected; therefore, the red color of the solution represents a composite of all colors transmitted (i.e., not absorbed) by the particles. To increasing the nanoparticles concentration the color change from light to dark red [50].

To prevent the NPs agglomeration all samples have been kept in glass vials in the fridge at 3 °C [29,31].

### 2.2. Morphological Characterizations of the Nanoparticles

In order to determine the morphology and the shape of the obtained colloidal NPs, for each sample, a drop of the colloidal solution has been casted on TEM grid and left drying in air. The analyses were carried out by using a TEM 2010 JEOL instrument (Jeol Ltd., Tokyo, Japan) operating at 200 KeV accelerating voltage and the images were analyzed by Gatan Digital Micrograph software to determine the NPs size distribution. For the samples obtained by 5 (5′ AuNPs) and 8 (8′ AuNPs) minutes of ablation time, the mean value of the NPs diameter, <D>, has been calculated on a statistical population of 300 NPs, the standard deviation on the mean value is associated as error. Instead, the nanostructures produced by 12 min of ablation time (12′ AuNPs) had not spherical shape but multifaceted (this caused by their agglomeration), so their average diameter has not been estimated.

### 2.3. Optical Characterizations of the Nanoparticles

To evaluated the position of LSPR and the stability of colloidal solutions a PerkinElmer Lambda 45 UV-Vis spectrophotometer (PerkinElmer, Inc., Waltham, MA, USA) was used, equipped with a halogen lamp for measurements in the VIS region and a deuterium lamp for measurements in the UV region, operating in the wavelength range of 190–700 nm with a resolution of ±0.1 nm.

For UV-Vis measurement the colloidal solutions were diluted in sodium citrate solution 0.1 mM by a factor of 1:10 directly inside the plastic cuvettes (optical path length 1 cm). The zero measurement (blank) was taken in citrate solution without colloids.

### 2.4. Glyphosate Sensing Test Through Ultraviolet-Visible (UV-Vis) Measurements with Analyte Sample

For the glyphosate sensing test, glyphosate (CAS number 1071836, analytical standard, www.sigmaaldrich.com) has been added to the colloidal solutions so to obtain solutions with decreasing concentration of glyphosate (2 mM, 1.8 mM, 1.5 mM, 1.2 mM, 1 mM, 0.5 mM, and 0.1 mM). In particular, a primary solution of glyphosate at concentration of 60 mM was prepared dissolving glyphosate powder in deionized Milli-Q water (resistivity 18 MΩ cm) and the analyte samples with different concentration of glyphosate were prepared directly in disposable plastic cuvettes. First, the cuvettes were filled with 0.2 mL of the NPs colloidal solution. Then, to each analyte sample was added a chosen volume of the primary solution previously prepared. After that, the cuvettes were filled with citrate solution 0.1 mM to a final volume of 2 mL in order to maintain the same concentration of Au NPs among the samples with and without glyphosate.

To ensure the reproducibility of the experiment, the whole processes and measurements were repeated about ten times over a time of six months.

## 3. Results and Discussion

**Figure 2:**
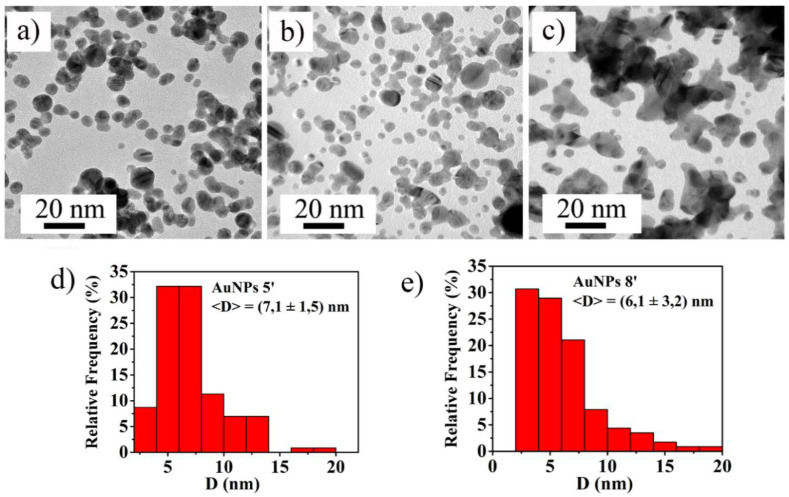
TEM images of synthesized 5′ Au nanoparticles (AuNPs) (**a**), 8′ AuNPs (**b**) and 12′ AuNPs (**c**). Diameters distribution of 5′AuNPs (**d**) and 8′ AuNPs (**e**).

The TEM images of the produced NPs are shown in Figure 2. In particular, for 5′ AuNPs (Figure 2a) and 8′ AuNPs (Figure 2b) it is easily visible that the nanoparticles have almost a circular section, indicating a three-dimensional spherical shape. Instead, the nanoparticles obtained from 12 min of ablation time present a multifaceted shape, indicating possible agglomeration process between the particles (see Figure 2c).

The size distributions of spherical NPs, displayed in Figure 2d for 5′ AuNPs and Figure 2e for 8′ AuNPs, reveal that most of them present a diameter in the range 3–7 nm, while for 12′ AuNP agglomerates exhibit a much wider size distribution, ranging from a few to tens of nm.

**Figure 3:**
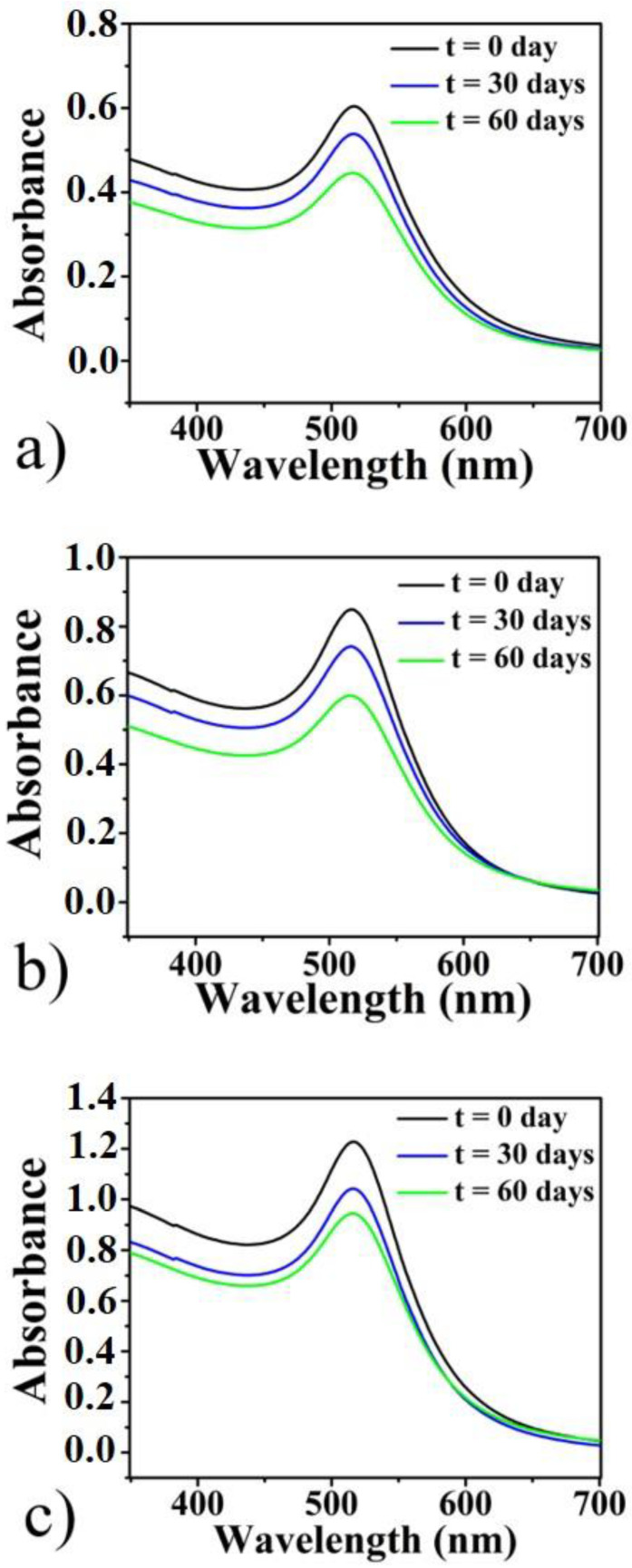
UV-Vis (Ultraviolet-Visible) absorbance spectra of laser synthesized Au nanocolloids immediately after the ablation process and 30 and 60 days after the production step. In particular, 5′ AuNPs (**a**), 8′ AuNPs (**b**), and 12′ AuNPs (**c**).

The colloids’ stability over the time was checked by UV–Vis spectrophotometry, the absorbance spectra were measured immediately after the ablation process, 30 and 60 days after the NPs production. As shown in Figure 3, all spectra have a LSPR lying in the visible region, centred at 516 nm and the peak intensity decreases over the days until it stabilizes after 60 days. In particular, after 60 days for 5′ AuNPs the intensity decreases by 13% (Figure 3a), for 8′ AuNPs by 19% (Figure 3b) and by 13% for 12′ AuNPs. This intensity decrease is explained by the occurrence of nanostructures aggregation process which is complete after about 60 days from the NPs production and resulting in stable solutions.

**Figure 4:**
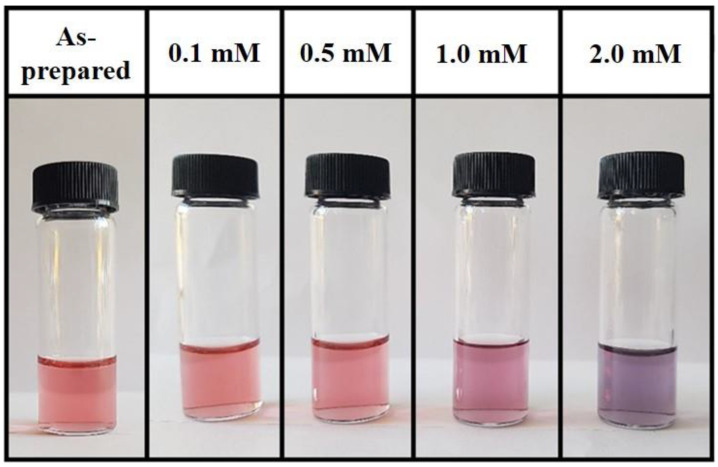
Picture of colloidal solutions with increasing glyphosate content from left to right.

Glyphosate at different concentrations was added to the colloidal solutions after 60 days from the colloidal solution preparation. Increasing the glyphosate concentration, the colors of the solutions change from red to purple. An example is shown in Figure 4: The as-prepared solution of 5′ AuNPs (on the left) presents a light red color which slightly changes by adding glyphosate and completely turns to purple at the highest concentration of 2 mM (on the right of the picture).

**Figure 5:**
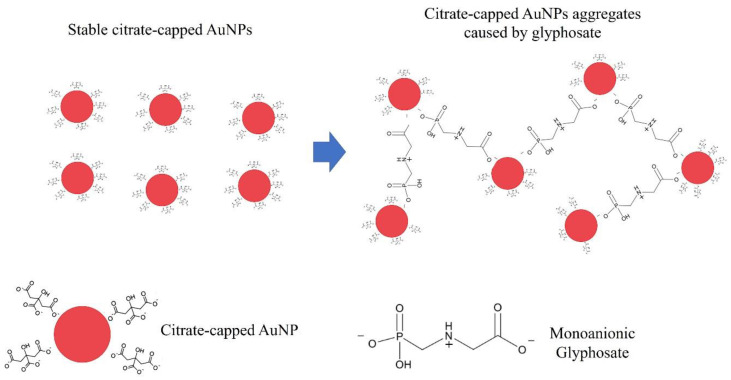
Schematic representation of the glyphosate detection strategy with citrate-capped AuNPs, not in scale.

The reason for this color change is shown in Figure 5: On the left side of the picture, the shown nanoparticles are surrounded by citrate anions, determining the formation of double layer on the surface of the nanoparticles, which by electrostatic repulsion avoids their aggregation and stabilizes the solutions. In the case of chemical synthesis of AuNPs by citrate reduction of auric acid, citrate molecules with negatively charged –COO^−^ groups cover the Au colloid, thus creating an NP with a highly negative-charged surface [53] determining the NPs electrostatic repulsion and the stabilization of the solutions. Similarly, Riabinina et al. [29] demonstrated, by Zeta potential measurements, that for laser ablated AuNPs in water in presence of citrate, a significant increase of Zeta potential (from −42 mV to −89 mV in their specific case) occurs with increasing the ablation duration. This indicates a strong interaction between freshly synthesized AuNPs and the surrounding solution and suggests that citrate molecules actively bond to the Au surface, similar to that found for Au colloids chemically prepared by citrate reduction method. In addition, the authors conclude that the variation in Zeta potential is most likely associated with the surface chemistry modification of the produced AuNPs in the sense the increase of the ablation time effectively results in the increase of the negative charge on the NPs surface. Regarding the effect of the glyphosate, first of all, we have to consider that an important point concerning the glyphosate interactions on surfaces is the molecule protonation states [54,55]: Glyphosate can form zwitterionic structures, which can be a monoanion (GLYP^−^) at a middle pH, which is our present case. Then, as schematically depicted in Figure 5, the mechanism of the AuNPs aggregation in presence of the monoanionic glyphosate, following, also, the considerations by De Góes et al. [46], can be qualitatively summarized as follows: Upon conversion of the zwitterion glyphosate to the monoanionic form, it can interact with the negatively charged AuNPs capped with citrate anions. In fact, the presence of the citrate anions attached to the AuNPs surface determines the formation of an electric double layer on the surface of the AuNPs, with the negative charges being the citrate anions attached to the AuNPs surface and a layer of positive charges on the surface of the AuNPs. In addition, the citrate anions attached to the AuNPs surface are easily displaceable by the monoanionic glyphosate molecule which can bind on the AuNP surface from a side and on the surface of another AuNP from the other side, and so on. The overall results is the decrease of the average AuNPs surface charge, which would break up the balance between electrostatic force and van der Waals’ force, leading to the aggregation of the citrate-capped AuNPs.

**Figure 6:**
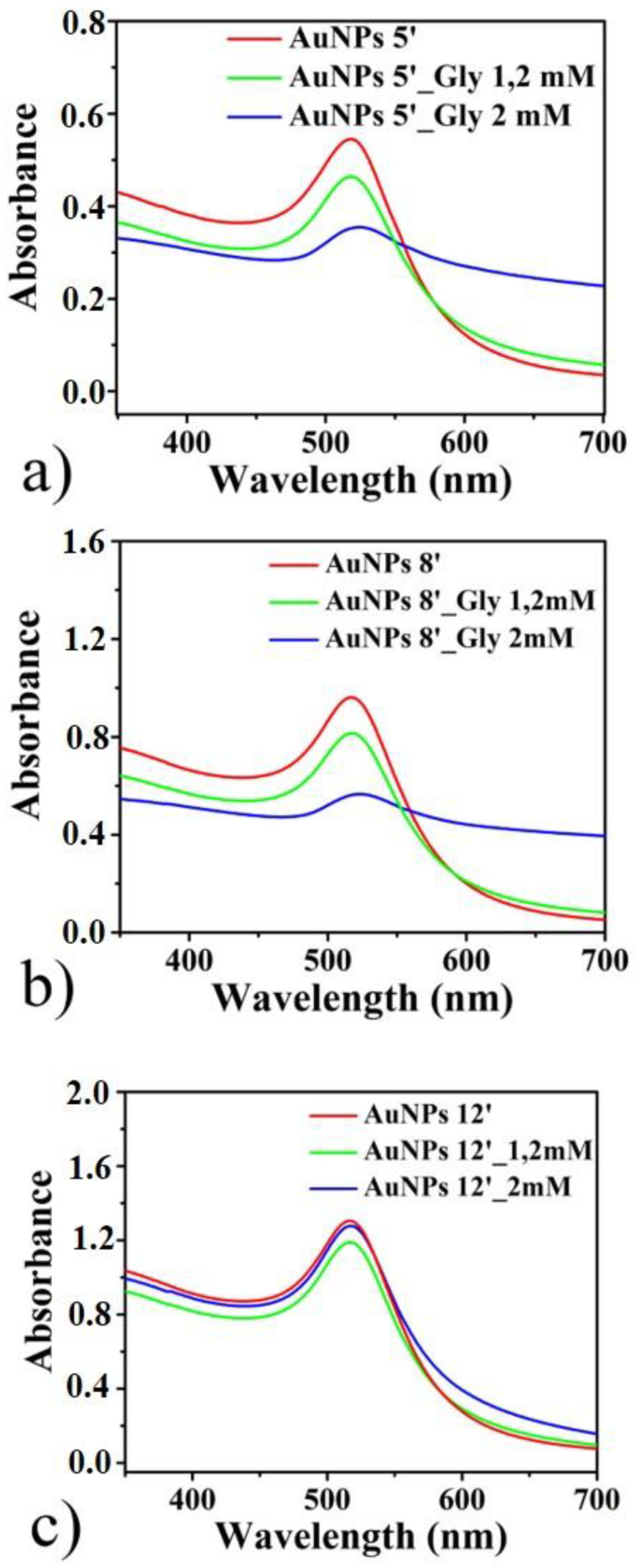
UV-Vis (Ultraviolet-Visible) absorbance spectra of 5′ AuNPs (**a**), 8′ AuNPs (**b**), and 12′ Au NPs(**c**) colloidal solution with different concentration of glyphosate.

This causes an appreciable change in the color of the solutions, phenomenon probably due to a hybridization of the plasmon modes of the AuNPs, resulting in higher energy (antibonding) or lower energy (bonding) plasmon modes [46,56,57]. As the concentration of glyphosate increases, more and more nanoparticles aggregate, in gradually larger nanostructures. This involves a change in the absorption properties of the colloidal solutions, which we have investigated by UV-Vis measurements. The obtained absorbance spectra are shown in Figure 6: (a) 5′ AuNPs, (b) 8′ Au NPs and (c) 12′ AuNPs. In the figure, it can be clearly seen that as the concentration of glyphosate increases, the intensity of the peak centered at 516 nm decreases and the component of the radiation absorbed at longer wavelength increases. Only the spectra of the solutions without glyphosate (red line), with glyphosate concentration of 1.2 mM (light blue line) and concentration 2 mM (blue line) are reported; the other spectra with different concentrations of glyphosate are not reported because they are very similar to those shown in the Figure 6.

**Figure 7:**
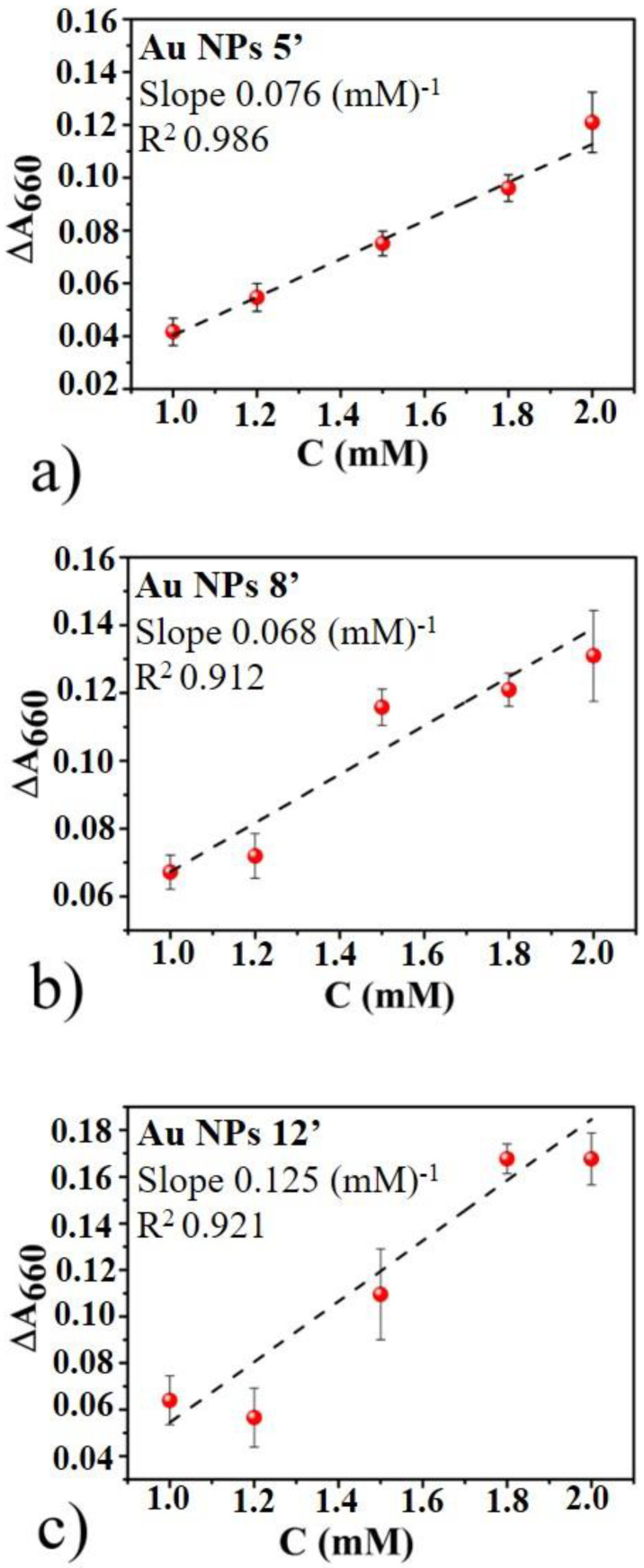
The plots of ΔA_660_ ($A_{\mathrm{with}\;\mathrm{glyphosate}}-A_{\mathrm{without}\;\mathrm{glyphosate}}$) at 660 nm at different glyphosate concentrations for 5′ AuNPs (**a**), 8′ Au NPs (**b**), and 12′ AuNPs (**c**).

For each sample, 10 different measurements were performed in order to increase the statistical population and, for each point, the standard deviation on the mean value is associated as error. From these data we extracted the colorimetric sensor calibration curves ΔA_660_ ($A_{\mathrm{with}\;\mathrm{glyphosate}}-A_{\mathrm{without}\;\mathrm{glyphosate}}$ at 660 nm) vs. glyphosate concentrations C (Figure 7), being ΔA_660_ = ($A_{\mathrm{with}\;\mathrm{glyphosate}}-A_{\mathrm{without}\;\mathrm{glyphosate}}$) at 660 nm with $A_{\mathrm{with}\;\mathrm{glyphosate}}$ the absorbance value corresponding to the wavelength of 660 nm for the colloidal solution containing glyphosate and $A_{\mathrm{without}\;\mathrm{glyphosate}}$ the absorbance value corresponding to the wavelength of 660 nm for the colloidal solution without glyphosate. The chosen wavelength value, λ = 660 nm, represents the wavelength of the diffused radiation for which there are the greatest differences, in the absorbance spectra, between the solutions with and without glyphosate and the wavelength value which ensures the best linearity in the calibration curves.

The plots in Figure 7a (5′ AuNPs), Figure 7b (8′ Au NPs) and Figure 7c (12′ AuNPs) show common trend: For glyphosate concentration values in the range from 1 mM to 2 mM the ΔA_660_ enhances with increasing concentration of glyphosate, exhibiting a linear correlation. The linear fits (dashed lines) of the experimental data (dots) allow to evaluate the sensitivity value for the glyphosate detection, represented by the slope of the straight line, the coefficient of linear determination R^2^ and the Limit of Detection (LOD). The LOD is obtained by 3.3 times the standard deviation of the y- intercept of regression line (S_y_), divided by the regression slope of the curve (S), LOD = 3.3(Sy/S) [47]. In particular, the LOD for 5′ AuNPs is 0.188 mM (31.7 mg/L); instead, it is 0.489 mM (82.6 mg/L) for 8′ AuNPs and 0.458 mM (77.4 mg/L) for 12′ AuNPs.

According these values, the produced Au colloidal solutions can be used as colorimetric sensors with a sensitivity on the glyphosate concentration detection of the order of 0.07 a.u./mM. The samples with the better linear trend are represented by Au 5′ NPs and 8′AuNPs colloidal solutions, this could be due to the presence of more monodisperse nanoparticles than the others solution (12′ AuNPs), as shown in Figure 2. In fact, since the Au 12′ NPs solution is composed of nanoparticles already partially aggregated (Figure 2c), the aggregation process due to the presence of glyphosate is less evident than that of the other two colloidal solutions because larger NPs could aggregate slower than the smaller NPs. In fact, the starting concentration of the Au NPs in the 12′ sample is much higher than in the 5′ and 8′ samples, due to the prolonged ablation time [31]. So, in the starting condition, the Au NPs are more closely spaced in the 12′ solution and the very short distance makes ineffective the citrate stabilizing effect. Hence, the unstable AuNPs aggregate, despite the effect of the capping agent and despite the increase of the negative Zeta potential on the surface of the NPs increasing the ablation time, resulting in the complex-morphology large Au aggregates recognized by Figure 2c. So, on the basis of the proposed aggregation mechanism of the AuNPs in presence of the glyphosate, the large Au structures observed in the 12′ sample, for each fixed concentration of the capping agent, are less and less reactive to the presence of glyphosate with respect to smaller AuNPs. This reduced reactivity results in a slower aggregation and, so, in a worst response to the presence of glyphosate.

## 4. Conclusions

In this work, citrate-capped AuNPs have been produced by PLAL at different ablation times. The NPs present almost a spherical shape and their average size is around 6 nm as estimated by TEM images. The stability of the solutions was evaluated by UV-Vis and the NPs are stable after 60 days. The Au colloidal solutions appear red and present a LSPR centered at 516 nm.

After the fabrication and the characterization, the colloidal solutions were used as colorimetric sensors to detect different concentrations of glyphosate.

In fact, the presence of citrate determines the formation of double layer on the surface of the nanoparticles stabilizing the solutions, instead, the presence of glyphosate determines electrostatic disorder on the surface of the Au nanoparticles and their aggregation in solution. As the concentration of glyphosate increases, more and more nanoparticles aggregate, in gradually larger nanostructures. This involves a change in the absorption properties of the colloidal solutions, in which the fraction of visible radiation absorbed at higher wavelengths increases, causing an appreciable change from red to purple in the color of the solutions.

The sensitivity on the glyphosate concentration was determined to be of the order of 0.07 a.u./mM. Also, the LOD for each solution was evaluated.

Based on these results, the use of gold nanoparticles, produced through a simple and low-cost method, allowed us to easily detect the presence of glyphosate without the use of advanced instruments. Anyway, this work can be considered a preliminary study, which in the next future will be expanded with selectivity tests (for example by testing the response to α-amino-3-hydroxy-5-methyl-4-isoxazolepropionic acid (AMPA)) and tests on real water samples will be checked. In fact, the permitted glyphosate levels in drinking water are lower than those found, for this reason we will improve the colloidal solutions, e.g., we will change the pH solution, the shape and morphology of the nanostructures, in order to increase the exposed surface of the detection structures for the analyte. Also, Zeta potential measurements for measuring the AuNPs surface charge are a key perspective of the present work to definitively support the proposed aggregation mechanism for the AuNPs in presence of glyphosate.

## References

[1] Maaz K. (2018). Silver Nanoparticles-Fabrication, Characterization and Applications.

[2] Johnston R.L., Wilcoxon J.P. (2012). Metal Nanoparticles and Nanoalloys.

[3] Garcia-Garcia F.J., Yubero F., Espinós J.P., González-Elipe A.R., Lambert R.M. (2016). Synthesis, characterization and performance of robust poison-resistant ultrathin film yttria stabilized zirconia—Nickel anodes for application in solid electrolyte fuel cells. J. Power Sources.

[4] Parra-Barranco J., García-García F.J., Rico V., Borrás A., López-Santos C., Frutos F., Barranco A., González-Elipe A.R. (2015). Anisotropic In-Plane Conductivity and Dichroic Gold Plasmon Resonance in Plasma-Assisted ITO Thin Films e-Beam-Evaporated at Oblique Angles. ACS Appl. Mater. Interfaces.

[5] Censabella M., Torrisi V., Compagnini G., Grimaldi M.G., Ruffino F. (2020). Fabrication of Metal Nanoparticles-Graphene Nanocomposites and Study of the Charge Transfer Effect. Physica E.

[6] Ruffino F., Crupi I., Simone F., Grimaldi M.G. (2011). Formation and evolution of self-organized Au nanorings on indium-tin-oxide surface. Appl. Phys. Lett..

[7] Ruffino F., Pugliara A., Carria E., Romano L., Bongiorno C., Fisicaro G., La Magna A., Spinella C., Grimaldi M.G. (2012). Towards a laser fluence dependent nanostructuring of thin Au films on Si by nanosecond laser irradiation. Appl. Surf. Sci..

[8] Ruffino F., Grimaldi M.G. (2014). Self-organized patterned arrays of Au and Ag nanoparticles by thickness-dependent dewetting of template-confined films. J. Mater. Sci..

[9] Ruffino F., De Bastiani R., Grimaldi M.G., Bongiorno C., Giannazzo F., Roccaforte F., Spinella C., Raineri V. (2007). Self-organization of Au nanoclusters on the SiO_2_ surface induced by 200 keV-Ar^+^ irradiation. Nucl. Instr. Meth. Phys. Res. B.

[10] Mishra Y.K., Kabiraj D., Sulania I., Pivin J.C., Avasthi D.K. (2007). Synthesis and characterization of gold nanorings. J. Nanosci. Nanotechnol..

[11] Mishra Y.K., Adelung R., Kumar G., Elbahri M., Mohapatra S., Singhal R., Tripathi A., Avasthi D.K. (2013). Formation of Self-organized Silver Nanocup-Type Structures and Their Plasmonic Absorption. Plasmonics.

[12] Mishra Y.K., Chakravadhanula V.S.K., Hrkac V., Jebril S., Agarwal D.C., Mohapatra S., Avasthi D.K., Kienle L., Adelung R. (2012). Crystal growth behavior in Au-ZnO nanocomposite under different environments and photoswitchability. J. Appl. Phys..

[13] Chakraborty U., Bhanjana G., Adam J., Mishra Y.K., Kaur G., Chaudhary G.R., Kaushik A. (2020). A flower-like ZnO-Ag_2_O nanocomposite for label and mediator free direct sensing of dinitrotoluene. RSC Adv..

[14] De M., Ghosh P.S., Rotello V.M. (2008). Applications of nanoparticles in biology. Adv. Mater..

[15] Lu A.H., Salabas E.E., Schüth F. (2007). Magnetic nanoparticles: Synthesis, protection, functionalization and application. Angew. Chem. Inter. Ed..

[16] Ghosh Chaudhuri R., Paria S. (2011). Core/shell nanoparticles: Classes, properties, synthesis mechanisms, characterization and applications. Chem. Rev..

[17] Monteiro D.R., Gorup L.F., Takamiya A.S., Ruvollo-Filho A.C., de Camargo E.R., Barbosa D.B. (2009). The growing importance of materials that prevent microbial adhesion: Antimicrobial effect of medical devices containing silver. Int. J. Antimicrob. Agents.

[18] Rostek A., Breisch M., Pappert K., Loza K., Heggen M., Köller M., Sengstock C., Epple M. (2018). Comparative biological effects of spherical noble metal nanoparticles (Rh, Pd, Ag, Pt, Au) with 4–8 nm diameter. Beilstein J. Nanotechnol..

[19] Homberger M., Simon U. (2010). On the application potential of gold nanoparticles in nanoelectronics and biomedicine. Philos. Trans. R Soc. London A.

[20] Maier S.A., Atwater H.A. (2005). Plasmonics: Localization and guiding of electromagnetic energy in metal/dielectric structures. J. Appl. Phys..

[21] Jain P.K., Huang X., El-Sayed I.H., El-Sayed M.A. (2008). Noble Metals on the Nanoscale: Optical and Photothermal Properties and Some Applications in Imaging, Sensing, Biology, and Medicine. Acc. Chem. Res..

[22] Li J., Zhao T., Chen T., Liu Y., Ong C.N., Xie J. (2015). Engineering noble metal nanomaterials for environmental applications. Nanoscale.

[23] Liu H.-L., Nosheen F., Wang X. (2015). Noble metal alloy complex nanostructures: Controllable synthesis and their electrochemical property. Chem. Soc. Rev..

[24] Maier S.A. (2007). Plasmonic: Fundamentals and Applications.

[25] Park J.-W., Shumaker-Parry J.S. (2014). Structural Study of Citrate Layers on Gold Nanoparticles: Role of Intermolecular Interactions in Stabilizing Nanoparticles. J. Am. Chem. Soc..

[26] Correard F., Maximova K., Estève M.-A., Villard C., Roy M., Al-Kattan A., Sentis M., Gingras M., Kabashin A.V., Braguer D. (2014). Gold nanoparticles prepared by laser ablation in aqueous biocompatible solutions: Assessment of safety and biological identity for nanomedicine applications. Int. J. Nanomed..

[27] Amendola V., Pilot R., Frasconi M., Maragò O.M., Iatì M.A. (2017). Surface plasmon resonance in gold nanoparticles: A review. J. Phys. Condens. Matter.

[28] Barcikowski S., Amendola V., Marzun G., Rehbock C., Reichenberger S., Zhang D., Gokce B. (2016). Handbook of Laser Synthesis of Colloids.

[29] Riabinina D., Zhang J., Chaker M., Margot J., Ma D. (2012). Size Control of Gold Nanoparticles Synthesized by Laser Ablation in Liquid Media. ISRN Nanotechnol..

[30] Barcikowski S., Compagnini G. (2013). Advanced nanoparticle generation and excitation by lasers in liquids. Phys. Chem. Chem. Phys..

[31] Censabella M., Torrisi V., Boninelli S., Bongiorno C., Grimaldi M.G., Ruffino F. (2019). Laser ablation synthesis of mono- and bimetallic Pt and Pd nanoparticles and fabrication of Pt-Pd/Graphene nanocomposites. App. Surf. Sci..

[32] Zeng H., Du X., Singh S.C., Kulinich S.A., Yang S., He J., Cai W. (2012). Nanomaterials via Laser Ablation/Irradiation in Liquid: A Review. Adv. Funct. Mater..

[33] Yang G. (2012). Laser Ablation in Liquids: Principles and Applications in the Preparation of Nanomaterials.

[34] Itina T.E. (2017). Laser Ablation-From Fundamentals to Applications.

[35] Sylvestre J.-P., Poulin S., Kabashin A.V., Sacher E., Meunier M., Luong J.H.T. (2004). Surface Chemistry of Gold Nanoparticles Produced by Laser Ablation in Aqueous Media. J. Phys. Chem. B.

[36] Zhang Q., Xu G., Gong L., Dai H., Zhang S., Li Y., Lin Y. (2015). An enzyme-assisted electrochemiluminescent biosensor developed on order mesoporous carbons substrate for ultrasensitive glyphosate sensing. Electrochim. Acta.

[37] Oliveira G.C., Moccelini S.K., Castilho M., Terezo A.J., Possavatz J., Magalhães M.R.L., Dores E.F.G.C. (2012). Biosensor based on atemoya peroxidase immobilized on modified nanoclay for glyphosate biomonitoring. Talanta.

[38] Stavra E., Petrou P.S., Koukouvinos G., Economou A., Goustouridis D., Misiakos K., Raptis I., Kakabakos S.E. (2020). Fast, sensitive and selective determination of herbicide glyphosate in water samples with a White Light Reflectance Spectroscopy immunosensor. Talanta.

[39] Da Silva Freire C., Moreno Santa Cruz R., Filho L.R.G., da Silva Moreira C., Falqueto A., de Medeiros E.S., de Souza Filho C.A., Valle A.L., do Nascimento Ferreira K. (2019). Application of a smartphone-based SPR platform for glyphosate detection. IEEE Sens. Appl. Symp..

[40] Cahuantzi-Munõz S.L., González-Fuentes M.A., Ortiz-Frade L.A., Torres E., Ţălu Ş., Trejo G., Méndez-Albores A. (2019). Electrochemical biosensor for sensitive quantification of glyphosate in maize kernels. Electroanal..

[41] Sok V., Fragoso A. (2019). Amperometric biosensor for glyphosate based on the inhibitionb of tyrosinase conjugated to carbon nano-onions in a chitosan matrix on a screen-printed electrode. Microchim. Acta.

[42] Viirlaid E., Ilisson M., Kopanchuk S., Mäeorg U., Rinken A., Rinken T. (2019). Immunoassay for rapid on-site detection of glyphosate herbicide. Environ. Monit. Assess..

[43] Ding X., Yang K.-L. (2013). Development of an oligopeptide functionalized surface plasmon resonance biosensor for online detection of glyphosate. Anal. Chem..

[44] Tu Q., Yang T., Qu Y., Gao S., Zhang Z., Zhang Q., Wang Y., Wang J., He L. (2019). In situ colorimetric detection of glyphosate on plant tissues using cysteamine-modified gold nanoparticles. Analyst.

[45] Zheng J., Zhang H., Qu J., Zhu Q., Chen X. (2013). Visual detection of glyphosate in environmental water samples using cysteamine-stabilized gold nanoparticles as colorimetric probe. Anal. Meth..

[46] De Góes R.E., Muller M., Fabris J.L. (2017). Spectroscopic Detection of Glyphosate in Water Assisted by Laser-Ablated Silver Nanoparticles. Sensors.

[47] De Góes R.E., Possetti G.R.C., Muller M., Fabris J.L. (2020). Tuning of Citrate-Stabilized Laser Ablated Silver Nanoparticles for Glyphosate Detection. IEEE Sens. J..

[48] Tan M.J., Hong Z.-Y., Chang M.-H., Liu C.-C., Cheng H.-F., Loh X.J., Chen C.-H., Liao C.-D., Kong K.V. (2017). Metal carbonyl-gold nanoparticle conjugates for highly sensitive SERS detection of organophosphorus pesticides. Biosens. Bioelectron..

[49] Vilela D., González M.C., Escarpa A. (2012). Sensing colorimetric approaches based on gold and silver nanoparticles aggregation: Chemical creativity behind the assay. A review. Anal. Chim. Acta.

[50] Mingos D., Michael P. (2014). Gold Clusters, Colloids and Nanoparticles I.

[51] Tarazona J.V., Court-Marques D., Tiramani M., Reich H., Pfeil R., Istace F., Crivellente F. (2017). Glyphosate toxicity and carcinogenicity: A review of the scientific basis of the European Union assessment and its differences with IARC. Arch. Toxicol..

[52] Clausing P., Robinson C., Burtscher-Schaden H. (2018). Pesticides and public health: An analysis of the regulatory approach to assessing the carcinogenicity of glyphosate in the European Union. J. Epidemiol. Community Health.

[53] Frens G. (1973). Controlled nucleation for the regulation of the particle size in monodisperse gold suspensions. Nat. Phys..

[54] Catão A.J.L., López-Castillo A. (2018). On the degradation pathway of glyphosate and glycine. Environ. Sci. Process. Impacts.

[55] Nafisah S., Morsin M., Jumadi N.A., Nayan N., Mohd Shah N.S., Razali N.L., An’Nisa N.Z. (2020). Improved Sensitivity and Selectivity of Direct Localized Surface Plasmon Resonance Sensor Using Gold Nanobipyramids for Glyphosate Detection. IEEE Sens. J..

[56] Polavarapu L., Mourdikoudis S., Pastoriza-Santos I., Pérez-Juste J. (2015). Nanocrystal engineering of noble metals and metal chalcogenides: Controlling the morphology, composition and crystallinity. CrystEngComm.

[57] Polavarapu L., Pérez-Juste J., Xu Q.-H., Liz-Marzán L.M. (2014). Optical sensing of biological, chemical and ionic species through aggregation of plasmonic nanoparticles. J. Mater. Chem. C.

